# A Digital, Self-Management Behavior Change Intervention for People With Chronic Obstructive Pulmonary Disease: Cohort Study

**DOI:** 10.2196/75683

**Published:** 2025-10-07

**Authors:** Liam Knox, Rachel Rahman, Gareth Norris, Carol-Anne Davies, Kimberley Littlemore, Claire Hurlin, Sam Rice, Keir Lewis

**Affiliations:** 1 Sheffield Institute for Translational Neuroscience, University of Sheffield, 385a Glossop Road, Sheffield, S10 2HQ, United Kingdom, 44 114 222 2230; 2 Department of Psychology, Aberystwyth University, Aberystwyth, United Kingdom; 3 Respiratory Department, Hywel Dda University Health Board, Carmarthen, United Kingdom; 4 Creative Department, eHealth Digital Media, Swansea, United Kingdom

**Keywords:** chronic obstructive pulmonary disease, self-management, telehealth, pulmonary rehabilitation, PocketMedic

## Abstract

**Background:**

People with chronic obstructive pulmonary disease (COPD) experience a range of limitations, which have a significant effect on their health. Self-management and pulmonary rehabilitation (PR) are key treatments for people with COPD; however, barriers often limit their uptake and adherence.

**Objective:**

To overcome these barriers, a digital self-management intervention called PocketMedic (PM) was developed and evaluated in people with COPD alongside, and in addition, to PR.

**Methods:**

A total of 53 participants were recruited to 1 of 3 groups: PM and PR, PM, or PR. Data were collected at baseline and 7 weeks (after the interventions had finished). Questionnaires on health-related quality of life, self-management knowledge, and disease knowledge were collected. Multivariate analysis of variances and ANOVAs were used to analyze the data.

**Results:**

The analyses found that the improvements in those receiving PM were not statistically significantly different from those receiving PR, indicating that PM may replicate the benefits underpinning self-management behaviors observed in those attending PR. However, there were no additional benefits when participants received PM and PR in combination.

**Conclusions:**

PM may be a useful treatment to support COPD self-management, especially when barriers prevent people with COPD receiving traditional services such as PR. The quantitative results suggest that PM may be less beneficial when delivered alongside PR. Feedback from participants indicated that they would prefer to receive PM while they were on the waiting list for PR, to support them during this time and alleviate the apprehensions associated with attending PR. Implications, limitations, and suggestions for future research are discussed.

## Introduction

People with chronic obstructive pulmonary disease (COPD) experience a range of daily-living limitations that have a significant effect on health-related quality of life (HRQoL) [1]. Regular symptoms include dyspnea, sputum production, coughing [2], as well as anxiety and depression [3]. Although people with COPD are extensive health care users [4], the majority of their time is spent outside of health services [5]. Therefore, knowing how to self-manage their condition is important for people with COPD; supporting regular self-management and increasing HRQoL have been identified as crucial objectives for health care providers [6].

For people with COPD, self-management behaviors include a range of activities such as regular exercise, taking medication, and attending health care appointments [7]; however, these behaviors are often neglected by people with COPD [1,8,9]. Self-management interventions combine educational components with behavior change techniques to engender long-term change [10] and have been associated with multiple beneficial outcomes, such as increased HRQoL and cost savings for health care providers [11-14]. Despite their effectiveness, these programs are often time-intensive for both people with COPD and health care staff [12,14-16].

Outside of specialist programs, self-management training is also a central component alongside structured, supervised exercise within pulmonary rehabilitation (PR) [17,18]. PR is a multidisciplinary group program recognized as one of the most effective interventions available for people with COPD [2,17,18]. However, attendance and adherence to PR are historically low [19-21], with a disruption to the usual routine, distance to the course, and a lack of transportation identified as common barriers [20]. In addition, access to PR is also a traditional problem, with some areas not able to provide the program [21,22].

To overcome the time-intensive and accessibility problems associated with current self-management and PR programs, this study used a digital, film-based, self-management intervention called PocketMedic (PM) and a parallel-group design to investigate its effectiveness in increasing self-management compared with, and in addition to, standard PR. This intervention is not intended to replace PR but is considered a potentially beneficial adjunct to the program, providing materials that people with COPD can use outside of PR, or an alternative when barriers restrict PR attendance. A diabetes version of PM has been used as an adjunct to health care previously, finding positive results [23]. Two hypotheses were formed: (1) people with COPD receiving solely PM would have increases in self-management knowledge, disease knowledge, and HRQoL, comparable to those receiving solely PR; and (2) people with COPD receiving PM in addition to PR would have larger increases in self-management knowledge, disease knowledge, and HRQoL, compared to those receiving PR or PM.

## Methods

### Study Design

This study used a parallel-group, cohort design. The project had 3 conditions: (1) people with COPD received both PM and PR (PM+PR), (2) only PM, or (3) only PR.

This study also measured the ability of PM and PR to increase self-determined, self-management motivation, alongside semistructured interviews. These findings have been published separately [24]; however, where qualitative feedback regarding PM was provided, this will be used to support the interpretation of this study’s findings within the discussion.

### Participants

Participants were recruited between September 2016 and March 2018 from Hywel Dda University Health Board (HDUHB) Wales, a semirural health region serving 3 counties, Carmarthenshire, Ceredigion, and Pembrokeshire. Consenting participants from Carmarthenshire who were due to attend PR were allocated to PM+PR or PR cohorts. Consenting participants from Ceredigion were allocated to PM (because PR was not available in this county at the time of this study). No one was recruited from Pembrokeshire.

Participants had to be eligible to attend PR, even if they did not receive this as part of their intervention. To attend PR for COPD within HDUHB, everyone must conform to the Global Initiative for Chronic Obstructive Lung Disease definition of COPD [2]: the participants must be ≥40 years old, have a 10 pack-year smoking history, and have spirometry less than 80% predicted. There were no exclusion criteria for the study.

**Figure 1.**
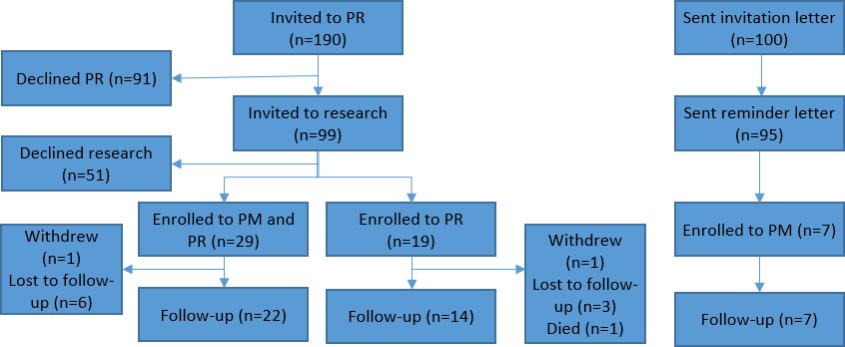
Number of participants at each stage of the study. PM: PocketMedic; PR: pulmonary rehabilitation.

Of the 55 people with COPD who consented, 2 withdrew from the study due to mental health reasons. Table 1 displays the demographics for the remaining 53 participants in the clinical trial. Figure 1 shows the flow of participants through the study.

**Table 1.** Participant demographics for each of the 3 research conditions.

Characteristics	PM^a^+PR^b^ (n=28)	PM (n=7)	PR (n=18)
Age (years), mean (SD)	66 (5.54)	68.1 (10.9)	65.3 (7.1)
Sex, n			
Male	15	6	11
Female	13	1	7
Smoking status, n			
Current smoker	3	2	1
Nonsmoker	3	0	0
Ex-smoker	15	5	14
Missing	7	0	3
Amount smoked per day (cigarettes), mean (SD)	20.3 (18.2)	26.4 (17)	25.4 (10.2)

a PM: PocketMedic.

b PR: pulmonary rehabilitation.

### PocketMedic

The PM is a digital, film-based intervention designed with the intention of increasing health outcomes in people with COPD by improving self-management motivation and knowledge. This is accomplished by delivering educational components and applying the principles of self-determination theory (SDT) [25,26] to better support people in engaging with their self-management. Specifically, techniques within the films were designed with the intention of increasing the satisfaction of the 3 basic psychological needs (autonomy, competence, and relatedness) by providing participants with meaningful rationales and choice, addressing obstacles for change, and identifying available support. A recent study has provided support for the use of SDT to predict outcomes in people with COPD [27].

A total of 10 digital films, lasting approximately 5 minutes each, could be watched by participants using a smartphone, tablet, or computer, at a time and place that suited them. The films showed a mix of health care professionals and people with COPD discussing the illness and providing information on how to self-manage effectively. PM covers a range of topics, including breathlessness, physical activity, and well-being. The films were available to participants for 7 weeks and could be rewatched at any time if desired. Multimedia Appendix 1 provides a title and brief description of each of the films used in this study.

### Pulmonary Rehabilitation

The study by Knox et al [28] describes in detail how HDUHB delivers PR, which is in line with best practice and current guidelines [17]. Briefly, approximately 7‐10 participants (per cohort) attended twice-weekly sessions for 7 weeks. An experienced occupational therapist, physiotherapist, and assistant exercise instructor delivered the course, which consisted of structured exercise for 1‐1.5 hours, followed by a 20‐ to 40-minute educational talk, which included topics such as smoking cessation, mental health, and dietary education.

### Data Collection

Participants completed questionnaires at baseline and at 7 weeks after the completion of PM+PR, PR, and PM. Questionnaires measured HRQoL, as assessed by the COPD assessment test[29]; self-management knowledge, as assessed by the Understanding COPD questionnaire (UCOPD) [30]; and disease knowledge, as assessed by the Bristol COPD Knowledge Questionnaire [31]. The COPD assessment test is negatively scored, with higher scores indicating a worse HRQoL. The UCOPD and Bristol COPD Knowledge Questionnaire are positively scored.

Adherence was operationalized as the number of PM films watched or PR sessions attended by participants. A maximum of 10 films could be viewed and 14 sessions attended.

All data were anonymized with participant-identifiable information replaced by a study number. Data were stored in a password-protected file, accessible only to members of the research team.

### Analysis

All quantitative data were analyzed using SPSS (IBM Corp). ANOVAs and chi-square tests were used to identify any possible baseline differences between the 3 research conditions. A multivariate analysis of variance (MANOVA) was conducted to test the hypotheses using the test statistic that maximized statistical power due to the relatively small sample size. In case of a significant MANOVA, separate ANOVAs for each dependent variable were implemented. Pairwise *t* tests, using the Sidak or Games-Howell correction, were used for post hoc analyses. All tests used an α level of .05.

Approximately half of the participants allocated to receive PM did not watch any of the digital health films. Therefore, subgroup analyses were conducted focusing solely on those who engaged with the intervention to remove any concealing effects nonengaging participants may have had on the results. Similar techniques have been used in previous self-management intervention evaluations [32]. The subgroup analyses results are presented in the Results section below to avoid confusion.

### Ethical Considerations

A favorable ethical opinion was provided by the Health Research Authority research ethics committee Wales 7 (16/WA/0130). The Declaration of Helsinki was followed throughout the study. All participants provided written informed consent. The participants’ privacy and confidentiality were maintained at all times. Funding was not available to compensate participants for their time.

## Results

### Overview

One-way ANOVAs and chi-square tests were conducted on participant demographics and baseline variables to identify any differences between participants in the 3 research conditions (Tables1  2).

**Table 2.** Baseline descriptive statistics for each of the 3 research conditions.

Measures	PM^a^+PR^b^ (n=28)	PM (n=7)	PR (n=18)
HRQoL^c^, mean (SD)	27.7 (6.21)	28.6 (3.60)	27.1 (5.15)
Self-management knowledge, mean (SD)			
About COPD^d^	69.6 (14.8)	59 (24.1)	66.1 (14.3)
Managing symptoms of COPD	63.1 (14.2)	53.5 (21.7)	53.7 (22)
Accessing help and support	43.2 (18.5)	38.2 (20.8)	44.4 (18.9)
Total	61.2 (10.3)	52.2 (17)	56.5 (15.2)
Disease knowledge	47.9 (14.9)	35.7 (8.50) *^e^	50.3 (9.59) *^e^

a PM: PocketMedic.

b PR: pulmonary rehabilitation.

c HRQoL: health-related quality of life.

d COPD: chronic obstructive pulmonary disease.

e * denotes statistically significant differences at *P*=.04.

Only the variable disease knowledge showed a significant difference between groups at baseline (*F*_2,38_=3.53; *P*=.04). Post hoc, pairwise *t* tests were implemented using the Games-Howell correction, finding statistically significant differences between participants receiving PM compared to those receiving PR (mean difference [MD] −14.5, 95% CI 0.51-28.6; *P=.*04). This indicates that, on average, those receiving PM alone had lower disease knowledge at baseline.

Table 3 displays average PM and PR, PM, or PR adherence for each of the 3 research conditions. As only 51.4% (18/35) of participants receiving PM+PR or PM watched one or more of the digital health films, average PM adherence (excluding nonengagers) was also calculated.

**Table 3.** Intervention adherence data for each of the 3 research conditions. Participants could watch a maximum of 10 PocketMedic films and attend 14 pulmonary rehabilitation sessions (depending on their research condition).

Intervention adherence	PM^a^+PR^b^ (n=28)	PM (n=7)	PR (n=18)
PM adherence, mean (SD)	2.93 (4.06)	7 (4.12)	—^c^
PM adherence (only engagers)^d^, mean (SD)	6.83 (3.35)	8.17 (2.99)	—
PR adherence, mean (SD)	10.33 (4.76)	—	9 (4.4)

a PM: PocketMedic.

b PR: pulmonary rehabilitation.

c Not available.

d For PM adherence (only engagers): n=12 for PM+PR; n=6 for PM.

To investigate whether access to PM was associated with increases in self-management knowledge, disease knowledge, and HRQoL, a repeated measures MANOVA was conducted. Using Pillai trace, there were no significant interaction effects between the 3 research conditions in any of the variables between baseline and follow-up (V=0.77, *F*_14,42_=1.89; *P*=.06). However, when the research condition was held constant, there was a significant effect for the within-group variable of time (V=0.81, *F*_7,20_=12.1; *P*<.001). The Bartlett test of sphericity was significant (*χ*^2^_27_=388; *P*<.001), and therefore the following ANOVAs used the Greenhouse-Geisser correction. The univariate tests found significant effects of time (between baseline and post) for all the included variables: HRQoL (*F*_1,26_=20.1; *P*<.001), UCOPD: about COPD (*F*_1, 26_=18.2; *P*<.001), UCOPD: managing symptoms of COPD (*F*_1,26_=10.3; *P=*.004), UCOPD: accessing help and support (*F*_1,26_=21.6; *P*<.001), UCOPD: total (*F*_1,26_=28; *P*<.001), and disease knowledge (*F*_1,26_=58.9; *P*<.001). All average scores at follow-up indicated more beneficial health states than those at baseline (Table 4).

**Table 4.** Baseline and follow-up descriptive statistics for all research measures for each of the 3 conditions.

Measures	PM^a^+PR^b^		PM		PR	
	Baseline	Follow-up	Baseline	Follow-up	Baseline	Follow-up
HRQoL^c^, mean (SD)	27.7 (6.43)	23.6 (4.99)	28.2 (4.32)	21.4 (3.91)	26.2 (5.57)	24.7 (6.75)
Self-management knowledge, mean (SD)						
About COPD^d^	66.8 (13.9)	76.5 (14.6)	65.1 (23.9)	72.9 (21.4)	65.3 (13.6)	68.6 (7.77)
Managing symptoms of COPD	60.3 (15.2)	66.5 (24.6)	47.4 (23.4)	66.7 (23.6)	54.4 (12.59)	62.5 (13.2)
Accessing help and support	49.5 (16.4)	56.7 (21.9)	34 (23.9)	62.5 (20.4)	41.8 (14)	59.8 (9.89)
Total	60.4 (11.3)	68.1 (17.1)	51.3 (20.2)	68.5 (21.3)	55.8 (6.93)	64.3 (7.53)
Disease knowledge	46.8 (15.2)	60.8 (19)	38.4 (8.74)	70.9 (6.22)	52.2 (8.68)	60.6 (9.04)

a PM: PocketMedic.

b PR: pulmonary rehabilitation.

c HRQoL: health-related quality of life.

d COPD: chronic obstructive pulmonary disease.

Unexpectedly, despite the original MANOVA indicating that there were no significant differences between the conditions, the follow-up ANOVAs showed a significant interaction effect between the conditions and time points for the variable of disease knowledge (*F*_2,26_=0.14; *P=*.003). However, pairwise, post hoc *t* tests using the Sidak correction found no significant differences between the research conditions (PM+PR vs PM: MD −0.88, 95% CI −17.2 to 15.4; *P*>.99; PM+PR vs PR: MD −2.63, 95% CI −15.6 to 10.3; *P=*.94; PM vs PR: MD −1.75, 95% CI −18.9 to 15.4; *P=*.99).

### Subgroup Analysis

The subgroup analysis excluding participants who did not watch any of the PM digital health films was repeated using the same MANOVA and ANOVAs described above.

Using Roy largest root, significant main effects were found for both time (*θ*=9.50, *F*_7,12_=16.3; *P*<.001) and research condition (*θ*=1.79, *F*_7,13_=3.33; *P=*.03). The Bartlett test of sphericity was again significant (*χ*^*2*^_27_=257; *P*<.001); therefore, the following ANOVAs used the Greenhouse-Geisser correction. Univariate analyses found significant differences between the 2 time points for all the included variables: HRQoL (*F*_1,18_=21.5; *P*<.001), UCOPD: about COPD (*F*_1,18_=14.8; *P*<.001), UCOPD: managing symptoms of COPD (*F*_1,18_=37; *P*<.001), UCOPD: accessing help and support (*F*_1,18_=46.8; *P*<.001), UCOPD: total (*F*_1,18_=71.2; *P*<.001), and disease knowledge (*F*_1,18_=44.7, *P*<.001)*.* All average scores at follow-up indicated more beneficial health states compared to those at baseline. Disease knowledge was the only variable to show a significant interaction effect between the 3 conditions and 2 time points (*F*_2,18_=6.02; *P*=.01), with HRQoL and self-management knowledge total outside the required level of significance (*F*_2,18_=3.31; *P*=.06 and *F*_2,18_=3.37; *P*=.06, respectively). However, post hoc, pairwise *t* tests conducted on the variable of disease knowledge using the Sidak correction again found no significant differences between the 3 research conditions (PM+PR vs PM: MD 2.60, 95% CI −17.7 to 22.9; *P=*.98; PM+PR vs PR: MD 0.85, 95% CI −16.4 to 18.1; *P>.*99; PM vs PR: MD −1.75, 95% CI −20.1 to 16.6; *P=*.99).

## Discussion

### Principal Findings

This study aimed to investigate whether a digital, film-based, self-management intervention could improve self-management knowledge, disease knowledge, and HRQoL in people with COPD, by comparing it to, and alongside, a well-established PR program. The quantitative analyses indicated that the improvements from those receiving PM were not significantly different from those receiving PR, resulting in the first hypothesis being accepted. This indicates that PM may replicate some of the benefits underpinning self-management behaviors seen by those attending PR. However, there were no additional benefits when participants received PM+PR, resulting in the second hypothesis being rejected.

The lack of statistically significant differences between those receiving solely PM and those receiving solely PR does support the potential to deliver PM independently. Travel and transport, in addition to a disruption to the usual routine, have been highlighted as barriers to PR adherence [20]. Participant feedback indicated that a particular strength of the intervention was the ability to watch the health films whenever and wherever the participant wished, in addition to being able to rewatch them if something was missed. The film format was also considered engaging and could provide information in a clearer manner than written leaflets. Therefore, the ability of a technology-delivered intervention to deliver significant benefits to self-management knowledge and understanding, potentially at par with PR, which can overcome these barriers, could prove beneficial [33]. This is supported by one of the key principles of the “A Healthier Wales” health care policy, being a reduction in geographical heterogeneity in the services provided [34]. Due to rurality and low staffing preventing PR from being delivered fully across HDUHB, PM may be a viable, effective, and suitable alternative, while recognizing the additional benefits that PR may offer through the structured, supervised exercise component.

There were some conflicting statistics associated with the variable of disease knowledge, with an original MANOVA in addition to post hoc *t* tests indicating no statistically significant differences between groups and an ANOVA reporting the reverse. Subsequently, the subgroup MANOVA and ANOVA found significant differences between the groups for disease knowledge, which were not replicated by the post hoc *t* tests. However, the analysis of baseline variables did indicate a significant difference between the groups for disease knowledge, where participants receiving PM had considerably lower levels of knowledge compared to the other 2 conditions. Therefore, it is possible that the contradictory statistics could be because of this baseline difference. Alternatively, the low sample size may have had an effect on the conflicting statistics; therefore, future research should replicate this study with a larger sample, in addition to using a randomized controlled trial design—the gold standard for evaluating the effectiveness of interventions.

The results of this study indicate that both PM and PR were effective at increasing self-management knowledge, disease knowledge, and HRQoL, corroborating previous literature regarding self-management interventions and PR [14,18,31,35]. However, the analyses showed that PM+PR did not result in a cumulative effect on the outcome measures. Although one possible interpretation is that there is a maximum level these variables can be improved in a 7-week period, collected feedback suggested that PM did not provide participants with anything additional to the PR learning, and, as a result, some participants stopped watching the health films altogether. Therefore, this feedback supports the interpretation that delivering PM alongside PR is not an effective way to distribute the health films. Several participants suggested that the interventions should be delivered independently of one another, with the consensus being that receiving PM before PR would be most beneficial. If delivered to people on a PR waiting list, this would enable people with COPD to receive self-management support at a time when they have limited treatment options, alleviating some of their concerns regarding the exercise components of PR. Fears of increased dyspnea due to exercise have been reported as a barrier to PR previously [36]; therefore, an intervention to reduce these fears may increase attendance. In addition, the UK’s PR audit found that 31% of those invited to PR failed to attend the initial assessment [21,37], representing the highest dropout throughout the entire program. Therefore, prescribing PM before PR could decrease this non-uptake and result in positive benefits for people with COPD and health care services.

Figure 1 shows the number of participants throughout the project and illustrates the lack of response from those invited to PR. Although a 31% nonuptake rate has been reported previously [37], this study found that 48% of people with COPD invited to PR did not attend the initial assessment. HDUHB serves a wide, rural geographical area, with only 1 out of its 3 counties regularly delivering PR. Therefore, given that travel and transport are both negative predictors of PR attendance and adherence [20], this could explain the heightened nonattendance rate. The acceptance rate for the research was also relatively low. Overall, 28% (55/199) of those invited consented to the study; however, there was only a 4% response rate for the PM condition, which used a postal consent process. This is significantly worse than the average of 20% reported widely in other studies using similar methods [38]. Although several strategies were implemented to increase the acceptance rate across the 3 research conditions, such as a 6-month extension, the sample size remains a limitation of this project, and therefore, the authors do suggest care in interpreting these results. However, one of the justifications for conducting this research in people with COPD is that self-management motivation in this condition is poor, evidenced by low adherence to treatment [39,40]. Therefore, although unfortunate, it is not surprising that there were recruitment issues within this study, because such issues are also evident throughout other areas of care for people with COPD [41]. Future research should explore how best to engage people with COPD with low motivation in both research and clinical practice.

This poor uptake was also found toward PM, with only 51.4% (18/35) of all participants who received the intervention watching any of the health films. However, when looking at each group individually, participants receiving PM+PR had an uptake rate of 42.9% (12/28), compared to 85.7% (6/7) for those receiving PM. In addition, on average, people receiving PM watched 80% (8/10) of the films, which is a higher adherence rate compared to the PR adherence for people receiving PM+PR (10/14, 71%) and those receiving PR (n/N, 64%). Therefore, participants who had access to another intervention (ie, PR) were less likely to engage with PM, whereas those who only received the health films had a high uptake and adherence rate. Thus, if PM is delivered independent of PR, the uptake and adherence may meet standard published thresholds of acceptability [42].

### Conclusions

In conclusion, this study provides novel contributions relevant to the care of people with COPD, such as the finding that although the interventions were effective separately, there were no cumulative benefits when the interventions were prescribed together. The lack of differences between the research groups does support the independent prescription of a technology-delivered, SDT-based, COPD self-management intervention, especially where environments prevent traditional programs from functioning fully. Participant feedback did support that prescribing PM to people with COPD before PR would be most beneficial. To the author’s knowledge, no previous study has evaluated an intervention that combines self-management, digital delivery, SDT, and people with COPD. Future research should investigate the safety and effectiveness of such an intervention within a larger sample size; however, this study does provide a proof of concept for increasing self-management knowledge, disease knowledge, and HRQoL in people with COPD through PM.

## Supplementary material

10.2196/75683 Multimedia Appendix 1 PocketMedic film titles.

## Abbreviations

COPD: chronic obstructive pulmonary disease
HDUHB: Hywel Dda University Health Board
HRQoL: health-related quality of life.
MANOVA: multivariate analysis of variance
PM: PocketMedic
PR: pulmonary rehabilitation
SDT: self-determination theory
UCOPD: Understanding COPD Questionnaire
