# Supplementary material for: A Digital, Self-Management Behavior Change Intervention for People With Chronic Obstructive Pulmonary Disease: Cohort Study
Source: JMIR Form Res. 2025 Oct 7;9:e75683. doi: 10.2196/75683 (PMC12505406; doi:10.2196/75683)
Supplement: Multimedia Appendix 1 [file formative-v9-e75683-s001.docx]

| Film title | Description |
| --- | --- |
| Introducing COPD | GP Dr Jane Gilbert explains COPD with the help of a sponge! Significant health improvements can be gained by making lifestyle changes, taking medication, and performing self-management. |
| Managing things yourself | Lawrence and Sandra share their experiences of living with COPD. Lawrence demonstrates some breathing exercises and shares practical advice. |
| Don’t talk to me about fitness | On the sofa, walking down the garden, or out with the dog – there are lots of ways to start exercising. Derrick and Alwyn know what it’s like to feel breathless and share their experiences, but the bottom line is that anything you can do will help. |
| Understanding breathlessness | “Breathlessness is the number one complaint and fear of those with COPD”. Dr Jane Gilbert acknowledges the anxiety among people with COPD. Susie remembers how she used to panic when she felt breathless but is pleased to report that she can now control it herself. |
| Welcome to pulmonary rehabilitation | A gentle introduction to a PR course and why it is so important. |
| Coping with COPD | “Half of the battle is your mental attitude”. People living with COPD share their strategies for coping with the ups and downs of daily life, from getting out in the car, to seeing friends, and surfing the internet. |
| Sleep | Alwyn, Susie, Derrick, and Lawrence talk about their experiences of sleeping and what works for them. |
| The proof is in the pudding | People with COPD and their families describe how a PR course has changed their lives. |
| COPD and medication | A whole armoury of medication is available to those affected by COPD. Dr Jane Gilbert talks about what is on offer and people living with COPD share their experiences. |
| Pace yourself | It can be hard to adjust to a new pace of life. Susie found that a PR course really made a difference to her everyday life. |
